# Phytanic acid stimulates glucose uptake in a model of skeletal muscles, the primary porcine myotubes

**DOI:** 10.1186/1476-511X-12-14

**Published:** 2013-02-11

**Authors:** Brita N Che, Niels Oksbjerg, Lars I Hellgren, Jacob H Nielsen, Jette F Young

**Affiliations:** 1 Department of Food Science, Aarhus University, Blichers Allé 20, Tjele, 8830, Denmark; 2 Department of System Biology, Technical University of Denmark, 2800 Kgs, Lyngby, Denmark; 3 Department of Food Science, Copenhagen University, 1958 Frederiksberg C, Copenhagen, Denmark

**Keywords:** Phytanic acid, Palmitic acid, Insulin, Glucose uptake, Glycogen synthesis, Viability, Primary porcine myotubes, Excess glucose, Free fatty acids

## Abstract

**Background:**

Phytanic acid (PA) is a chlorophyll metabolite with potentials in regulating glucose metabolism, as it is a natural ligand of the peroxisome proliferator-activated receptor (PPAR) that is known to regulate hepatic glucose homeostasis. This study aimed to establish primary porcine myotubes as a model for measuring glucose uptake and glycogen synthesis, and to examine the impact of physiological amounts of PA on glucose uptake and glycogen synthesis either alone or in combination with insulin.

**Methods:**

Porcine satellite cells were cultured into differentiated myotubes and tritiated 2-deoxyglucose (2-DOG) was used to measure glucose uptake, in relation to PA and 2-DOG exposure times and also in relation to PA and insulin concentrations. The MIXED procedure model of SAS was used for statistical analysis of data.

**Results:**

PA increased glucose uptake by approximately 35%, and the presence of insulin further increased the uptake, but this further increase in uptake was non- additive and less pronounced at high insulin concentrations. There was no effect of PA alone on glycogen synthesis, while the insulin stimulation of glycogen was increased by 20% in the presence of PA. PA neither stimulated glucose uptake nor glycogen synthesis in insulin-resistant myotubes generated by excess glucose exposure.

**Conclusions:**

Primary porcine myotubes were established as a model of skeletal muscles for measuring glucose uptake and glycogen synthesis, and we showed that PA can play a role in stimulating glucose uptake at no or inadequate insulin concentrations.

## Background

Due to its high concentration of saturated fatty acids, dairy fat intake has been associated with increased risk of cardiovascular diseases and type 2 diabetes in humans [1,2]. However, recent meta-analysis of data from prospective cohort-studies shows an inverse association between intake of dairy products and the incidence of both cardiovascular diseases and type 2 diabetes [3], and available data do not indicate any increased risk of high-fat dairy products compared to low-fat dairy products [4]. Similarly, studies using the odd-chained fatty acids C15:0 and C17:0 as validated biomarkers of dairy fat intake showed that a higher intake of dairy fat was associated with a decreased risk of myocardial-infarction and development of the metabolic syndrome [5-8]. These findings suggest the existence of a milk-fat paradox; despite the saturated profile of milk-fat, it does not seem to increase metabolic risks, but might actually be protective. Hence, further research is necessary to elucidate this controversy and to identify potential components in dairy fat that might be responsible for the protective effect.

Milk is a very rich source of potentially bioactive lipids such as short-chained fatty acids and conjugated linoleic acid (CLA) [9,10]. Another fatty acid of interest in milk is phytanic acid (PA); a C20 saturated fatty acid with four methyl-branches, which is known to be a natural ligand of the nuclear receptors peroxisome proliferator- activated receptors (PPAR) and retinoid-X- receptors (RXR) [11-13]. The PPARs are lipid sensors, which act by regulating body glucose and lipid homeostasis [14], and it has been shown that PA enhances glucose uptake in rat hepatocytes, possibly in a PPAR-dependent manner, at least at high concentrations [15]. Based on these observations, it was suggested that PA might improve glucose- homeostasis, and thereby protect against the development of the metabolic syndrome [16]. Dairy products or meat from ruminants are major sources of PA in the human diet [17]. The concentration of PA in plasma from healthy humans vary between 0.04 and 11.5 μM [18], and is strongly dependent on the intake of ruminant products [19].

Skeletal muscle fibers are the major site for insulin-regulated glucose uptake, accounting for over 70% of glucose disposal after a meal [20]. Furthermore, excess glucose and free fatty acid exposure lead to insulin resistance in these muscle fibers [21,22]. Hence, effects of PA on skeletal muscle glucose metabolism are expected to have an impact on total glucose homeostasis at the organism level. The main aim of this study was therefore to establish primary porcine myotubes as a model for glucose uptake and glycogen synthesis, and to examine whether physiological concentrations of PA can alter glucose uptake and the rate of glycogen synthesis in primary porcine myotube cultures, either alone or in combination with insulin.

## Results

### Viability assay

Primary porcine myotubes were incubated with doses of PA ranging from 1–500 μM (Table 1). The viability of the myotubes was not affected by 1–10 μM PA. Exposure of the myotubes to 20 μM PA markedly reduced their viability by about 20%. This viability dropped by 36% after exposure to 500 μM PA. For comparative reasons, the effect of palmitic acid (PAM) on viability was also assayed (Table 1). Exposure of myotubes to 1 or 10 μM PAM had no effect on viability while 50 μM PAM reduced their viability by about 15% and by 500 μM PAM the viability of the myotubes were reduced to only 54%.

**Table 1:** Effect of fatty acids on the viability of primary porcine myotubes

***Fatty acid concentration (μM)***	***Fold change in viability***	
	***PA***	***PAM***
Control (0)	1^a^ ± 0.023	1^a^ ± 0.036
1	1.01^a^ ± 0.030	
10	0.97^a^ ± 0.022	0.95^a^ ± 0.031
20	0.81^b^ ± 0.036	
50	0.74^bc^ ± 0.042	0.85^b^ ± 0.032
100	0.70^c^ ± 0.046	0.82^b^ ± 0.035
200		0.74^c^ ± 0.029
300		0.61^d^ ± 0.051
500	0.64^c^ ± 0.025	0.54^d^ ± 0.030

Differentiated porcine myotubes were incubated with different concentrations of fatty acids (PA or PAM) for 24 h, and then treated with WST-1 reagent as mentioned in the methods. Control samples contained experimental media without fatty acids. Data are expressed as LSmean values from five-eight replicate wells, carried out in three separate experiments with cells isolated from a different pig each time. LSmeans with different letters (a-d) within each row denote significantly different responses related to fatty acid treatments; (P < 0.05).

### Optimization in relation to glucose uptake assay

**Figure 1:**
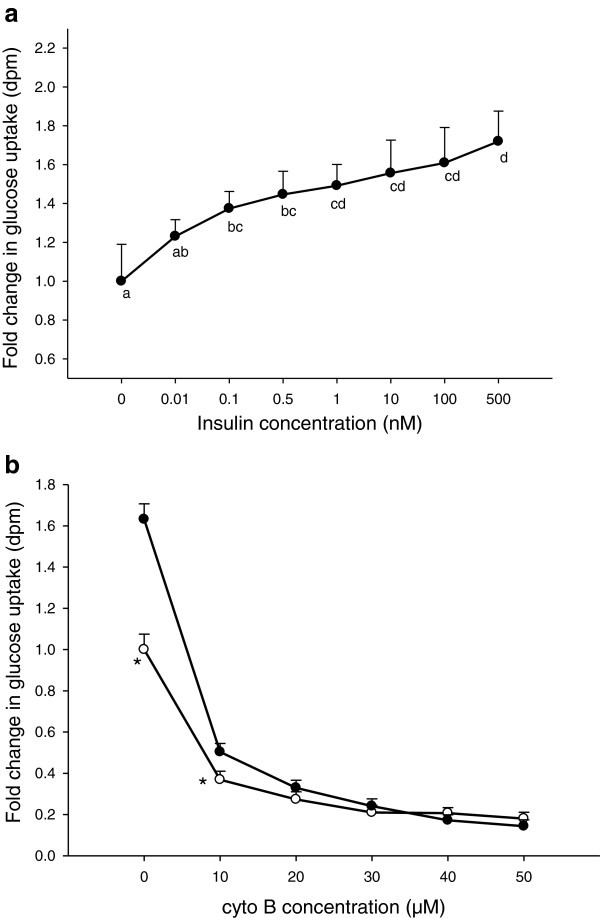
**Glucose uptake in porcine myotubes in response to insulin and cyto B.** Glucose uptake was performed on differentiated porcine myotubes in the presence of varying concentrations of insulin (**a**), or varying concentrations of cyto B with (full circles) or without (open circles) 500 nM insulin (**b**). Control samples lacked cyto B. Data are expressed as LSmean values of four replicate wells, carried out in at least two separate experiments with cells isolated from a different pig each time. * denotes LSmeans with significant difference in responses with or without insulin; (P < 0.05).

Glucose uptake in primary porcine myotubes was insulin-dose-dependent (Figure 1a). At 0.1 nM insulin, a significant increase in glucose uptake was recorded and at a supraphysiological concentration of 500 nM insulin, a 70% increase in uptake was obtained. To verify the presence of carrier-mediated glucose uptake in the primary porcine myotube model, different doses of cytochalasin B (cyto B) (0–50 μM) were added to the differentiated myotubes (Figure 1b). At 10 μM cyto B, glucose uptake in myotubes with and without insulin was reduced to 54 and 40%, respectively, when compared to that of control cells without insulin or cyto B addition. In the presence of 20–50 μM cyto B, the glucose uptake was reduced to approximately 70-90% of controls without cyto B, irrespective of insulin addition.

### Glucose uptake as a function of PA incubation time and 2-DOG incubation time

**Figure 2:**
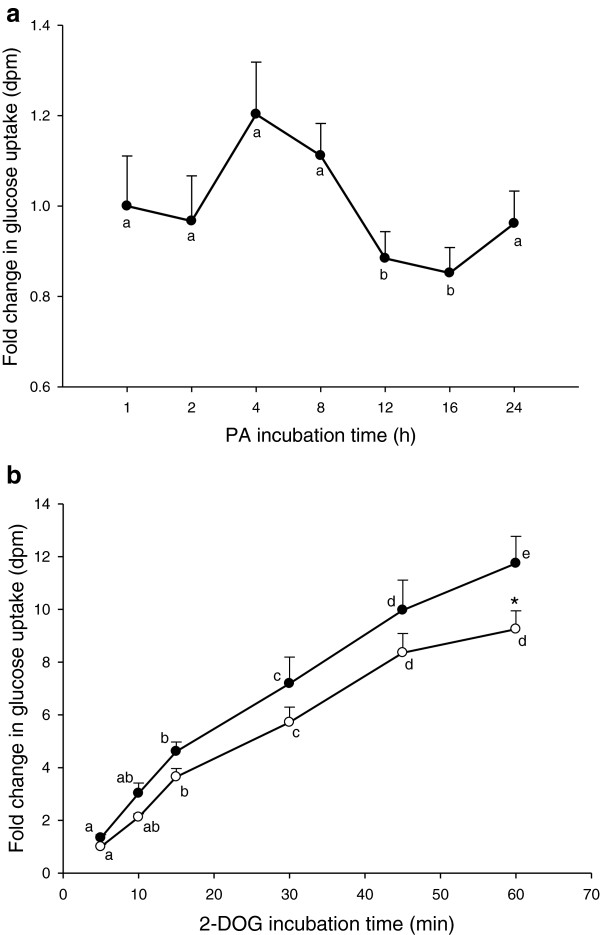
**Glucose uptake with or without PA as a function of 2-DOG incubation time.** Glucose uptake was performed on differentiated myotubes that had been incubated with (**a**) 10 μM PA for different times and then treated with 2-DOG for 30 min, or (**b**) with (full circles) or without (open circles) 10 μM PA for 4 h and afterwards treated with 2-DOG for 5–60 min. Data are expressed as LSmean values of triplicate wells carried out in three separate experiments with cells isolated from a different pig each time. LSmeans with different letters (a-e) denote significantly different responses related to incubation times, while * denotes LSmeans with significantly different responses with or without PA; (P < 0.05).

Based on the results from the viability studies and the concentration profile of PA in human plasma, glucose uptake assays were performed in myotubes that were incubated with 10 μM PA over a period of 24 h to define an effective incubation time with PA (Figure 2a). No significant increase in glucose uptake was observed after 1 h incubation with PA. There was, however, a tendency of elevated glucose uptake between 4–8 h of incubation with PA followed by a temporary marked decrease between 12–16 h of incubation, when compared to 1 h of incubation.

To estimate an optimal incubation time for 2-DOG to achieve effective uptake, myotubes were exposed to 2-DOG for 5–60 min with or without 10 μM PA (Figure 2b). There was a general steady increase in glucose uptake with increasing time of incubation with 2-DOG. The steepest increase in 2-DOG uptake was observed during the first 15 min, while the process was slowest during the last 10 min of incubation. In the presence of PA, glucose uptake tended to be higher at all the different incubation times, but this difference was significant only after 60 min of incubation with 2-DOG.

### Glucose uptake in response to PA and PAM

When testing the effect of PA on glucose uptake (Table 2), a 23% increase in uptake was noted even at the sub-physiological concentration of 0.5 μM PA. Increasing the PA concentration further did not significantly increase the uptake, although average uptake increased to between 30-35%. The effect of 1–10 μM PA could not be mimicked by same concentrations of PAM (Table 2). However, when myotubes were exposed to higher doses of PAM, there were marked changes in glucose uptake. Glucose uptake dropped to 75% when the myotubes were exposed to 100 μM PAM and at 200 μM and 400 μM PAM, the glucose uptake ability of the myotubes was only 50% and 30%, respectively, when compared to controls.

**Table 2:** Glucose uptake in response to PA and PAM

***Fatty acid concentration (μM)***	***Glucose uptake***	
	***PA***	***PAM***
Control (0)	1.00^a^ ± 0.045	1.00^a^ ± 0.067
1	1.23^b^ ± 0.105	1.09^a^ ± .051
5	1.29^b^ ± 0.071	
10	1.32^b^ ± 0.075	1.14^a^ ± 0.061
20	1.34^b^ ± 0.166	
40	1.37^b^ ± 0.076	
50		0.92^a^ ± 0.032
60	1.37^b^ ± 0.079	
100		0.75^b^ ± 0.032
200		0.48^c^ ± 0.04
400		0.32^d^ ± 0.028

Glucose uptake was carried out on differentiated myotubes that had been incubated with 1–60 μM PA or 1and 10 μM PAM for 4 h. PAM from 50–400 μM was administered for 24 h. Control samples lacked fatty acids. Data are expressed as LSmean values of triplicate wells, carried out in three separate experiments with cells isolated from a different pig each time. LSmeans with different letters (a-d) within each row denote significantly different responses related to fatty acid treatments; (P < 0.05).

### Glucose uptake and glycogen synthesis with or without PA as a function of insulin

**Figure 3:**
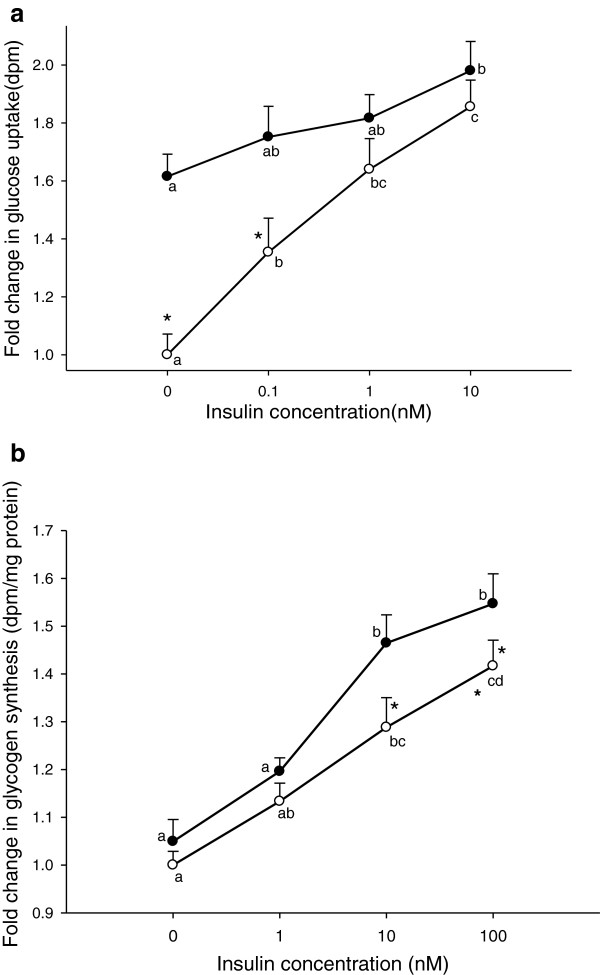
**Glucose uptake and glycogen synthesis with or without PA as a function of insulin.** Glucose uptake (**a**) was carried out on differentiated myotubes that had been incubated with (full circles) or without (open circles) 10 μM PA for 4 h, followed by different concentrations of insulin during the last 1 h. Glycogen syntheses (**b**) were performed on myotubes treated with (full circles) or without (open circles) 10 μM PA for 4 h, followed by 0–100 nM insulin for 2 h. Control samples lacked PA and insulin. Data are expressed as LSmean values collected from three-four wells, carried out in three separate experiments with cells isolated from a different pig each time. LSmeans with different letters (a-d) denote significantly different responses related to the insulin concentration, while * denotes LSmeans with significantly different responses with or without PA; (P < 0.05).

The impact of PA on glucose uptake and glycogen synthesis was also studied in relation to various insulin concentrations. Addition of 10 μM PA caused a further increase in glucose uptake at all levels of insulin (Figure 3a), but a significant improvement was only achieved at lower insulin concentrations (0 and 0.1nM). The incorporation of glucose into glycogen increased in response to insulin treatment from 0–100 nM, with a significant increase at 10 and 100 nM insulin (Figure 3b). Overall, glycogen synthesis was not significantly affected by PA alone, regardless of the increased tendency. However, in the presence of 10 nM insulin, PA caused an additional increase in glycogen synthesis of about 20%.

### Exposure of myotubes to extracellular glucose

**Figure 4:**
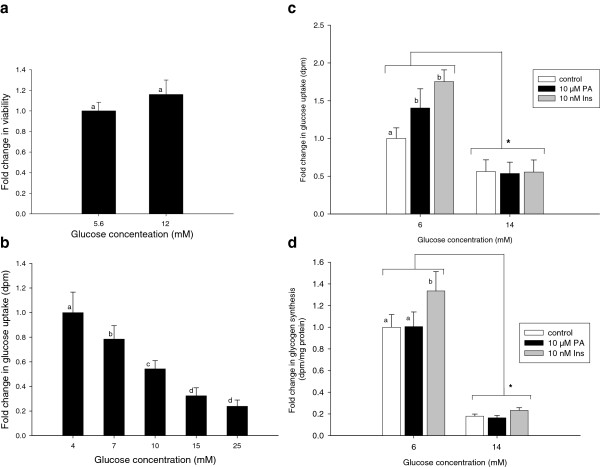
**Exposure of myotubes to extracellular glucose.** Differentiated myotubes treated with two different concentrations of glucose for 24 h were subjected to viability assay (**a**). Glucose uptake was also performed on myotubes exposed to various glucose concentrations (**b**). Myotubes exposed to 6 mM glucose (controls) or 14 mM glucose for 24 h in the absence (open bars) or presence of PA (black bars) or insulin (grey bars) were subjected either to (**c**) glucose uptake or (**d**) glycogen synthesis measurements. PA was administered for 24 h while insulin was given during the last 1 h of incubation for glucose uptake measurements and 2 h for glycogen synthesis. Data are expressed as LSmean values of four replicate wells, carried out in three separate experiments with cells isolated from a different pig each time. LSmeans with different letters (**a** and **b**) denote significantly different responses related to PA or insulin treatments, while statistical difference in LSmeans between controls and myotubes given 14 mM glucose is denoted with *; (P < 0.05).

The viability of the myotubes after exposure to excess glucose (12 mM) was not significantly affected (Figure 4a). The addition of 7, 10 and 15 mM of extracellular glucose reduced glucose uptake to about 80, 65 and 23%, respectively, when compared to myotubes exposed to only 4 mM of extracellular glucose (Figure 4b). Higher amounts of extracellular glucose did not cause further drops in glucose uptake. While 10 μM PA and 10 nM insulin caused an approximate 40 and 75% increase, respectively, in glucose uptake in myotubes exposed to 6 mM extracellular glucose, no effect of neither PA nor insulin was observed when myotubes were exposed to 14 mM extracellular glucose (Figure 4c). Glycogen synthesis was reduced to 25% when myotubes were treated with 14 mM glucose, compared to myotubes treated with 6 mM glucose (Figure 4d). The marked effect of 10 nM insulin on glycogen synthesis in the presence of 6 mM glucose was abolished by exposing the myotubes to 14 mM glucose (Figure 4d).

## Discussion

**Figure 5:**
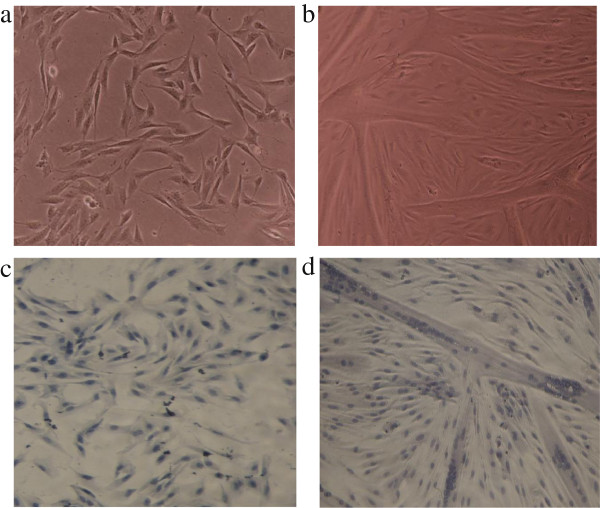
**Microscopic view of proliferating and differentiating porcine satellite cells.** Proliferating myoblasts at 80% confluence (**a**) or myotubes at day 2 of differentiation (**b**) were stained as described in the methods to reveal the single nuclei cells of myoblasts (**c**) and multi-nuclei tubular structure of myotubes (**d**). Picture magnification was 100 x.

Phytanic acid (PA) is a natural ligand and activator of the PPARs, specifically PPAR-α and γ [23]. The PPARs have been implicated not only in the regulation of adipose tissue development and insulin signaling [24] but also in modulating insulin sensitivity of muscles [25,26] and increasing exercise endurance [27]. It has been hypothesized that PA can function as a natural PPAR agonist and therefore, be useful in the prevention and treatment of diabetes [28]. Hence, PA may have a positive influence on the regulation of glucose metabolism. As skeletal muscle is the major site of insulin-dependent glucose uptake [20], we established a primary myotube model to study the effect of PA on glucose-homeostasis. The porcine primary myotube model shares many similarities to the human myocytes and has fewer artifacts when compared to immortal cell line systems [29]. Satellite cells were grown into myoblasts (Figure 5a and c) and successfully differentiated into characteristic multinucleated myotubes (Figure 5b and d), just as in previous findings [30].

Initially, the suitability of the primary porcine myotube model was tested, and assay conditions optimized in relation to both glucose assay compound (2-DOG) and PA exposure. Viability assays were performed to determine an appropriate concentration of PA that did not compromise the cells metabolic activities. The viability of the myotubes was noticeably reduced when exposed to concentrations of PA equal to or above 20 μM (Table 1). The toxic effect of PA at 20 μM or above may be attributed to apoptosis of the cells, as shown previously by studies in human skin fibroblasts and rat liver [31,32]. The average concentration of PA in human plasma has been reported to be between 0.04-11.5 μM [18,19,33], with higher values found predominantly in meat eaters and dairy product consumers [18,19]. Based on this, and the results from our viability studies, the working concentration of PA was chosen as 10 μM. Palmitic acid (PAM); a fatty acid of same chain length as PA but without the branching methyl-groups was used as a control, and we showed that 1 and 10 μM PAM had no effect on myotube viability. At concentrations of 50 μM PAM and above, the viability of the myotubes were reduced, possibly in a manner similar to that of higher amounts of PA [31,32].

The non-metabolizable analogue of glucose; 2-deoxyglucose (2-DOG), is a suitable marker for the measurement of muscle glucose transport as it can be transported into cells just like glucose [34], and it was used in this study to measure glucose uptake. Glucose transport in muscles is insulin-dependent and mediated by GLUT-4 transporters, as well as insulin-independent, mediated by GLUT-1 transporters [35]. Insulin treatment of varying concentrations caused a dose-dependent increase in glucose uptake in the myotubes (Figure 1a), proving that the primary porcine myotubes have a functional insulin-stimulated GLUT-4 translocation to the plasma membrane [36]. The action of both GLUT-1 and GLUT-4 in the carrier-mediated glucose uptake was checked by treatment of the myotubes with cytochalasin B (cyto B); a drug known to inhibit glucose transport by binding to glucose transporters with a higher affinity than glucose [37,38]. From our observation, cyto B inhibited both the insulin- and non-insulin-mediated glucose uptake to a similar magnitude in primary porcine myotubes (Figure 1b). The reduction in glucose uptake by cyto B confirms that both the insulin- and the non-insulin-mediated transport systems are functional in our primary porcine myotube model. Our observation with cyto B also showed that about 20% of glucose uptake in the myotubes was not affected by cyto B. One can speculate that a minimal steady state of non-specific glucose uptake is maintained in the porcine cultures. It is also likely that other GLUT receptors than GLUT 1 and 4 are present in the myotubes [39], and cyto B cannot bind to them. In that case glucose transport is possible even in the presence of cyto B.

A biphasic effect on glucose uptake was observed during the 24 h incubation of myotubes with 10 μM PA (Figure 2a). Since only a subtle increase in glucose uptake was noticed after 4 and 8 h exposure to PA, it is less likely that the subsequent fall in glucose uptake is due to depletion of GLUT depots. Other unknown factors could, thus, be responsible for this drop in glucose uptake. A rise in glucose uptake at 24 h could be linked to consequent up-regulation of transcripts responsible for the translation of proteins that skew up glucose uptake.

Glucose uptake has been shown to be linear for over 20 min in a human skeletal muscle cell line study [40], and in the present study, the steepest increase in glucose uptake was observed during the first 15 min of incubation with 2-DOG and by 45 min, the curve started to level off (Figure 2b). It is possible that after 45 min incubation with 2-DOG, a partial saturation of the normal glucose pathway is attained. Following these observations, the incubation time with 2-DOG was set at 30 min.

Considering the effects of PA on glucose uptake, it is noteworthy that a concentration as low as 1 μM caused a 25% increase in glucose uptake. Increasing the PA concentration to 10 μM slightly increased glucose uptake further (Table 2), while equivalent amounts of PAM had no effect on glucose uptake. The effect of PA was, thus, a specific effect, which is in agreement with a similar study in rat hepatocytes, in which 100 μM PA markedly increased glucose uptake following incubation with 2-DOG, when compared to docosahexaenoic acid [15]. The reduction in glucose uptake observed at PAM concentration of over 100 μM (Table 2) was partly due to inhibition of glucose transport activity [41], but also most likely a consequence of stress [31].

The effect of PA diminished with increasing insulin stimulation of glucose uptake (Figure 3a). This could indicate a similar mode of action between insulin and PA. Our results also indicate that the PA concentrations normally found in plasma (> 1 μM), are sufficient to enhance non-insulin dependent glucose uptake and that alteration of plasma PA concentrations within normal physiological range, only have minor effects. Hence, we suggest that normal levels of PA is a relevant factor in controlling fasting glucose homeostasis *in vivo* and that increased intake of PA only will affect glucose uptake in subjects with extremely low PA-levels (for example strict vegetarians with no intake of dairy products or ruminant meat).

Despite the fact that 10 μM PA induced an increased glucose uptake in the absence of insulin (Figure 3a), it did not enhance the rate of glycogen synthesis (Figure 3b). Thus, PA at physiological concentrations increases glucose uptake in the absence of insulin, without causing a concomitant increase in glycogen synthesis, why the absorbed glucose must be shunted into other metabolic pathways. However, the combination of 10 μM PA and 10 nM insulin more than doubled the rate of glycogen synthesis, compared to the effect of insulin alone (45% vs. 20%). This creates a somewhat paradoxical situation that increasing insulin concentrations attenuate the effect of PA on glucose uptake, but enhance its effect on glycogen synthesis. Hence, the two effects must be explained by different mechanisms of action of PA. It should be noted, that the effect of PA on both glucose uptake and glycogen synthesis is not a general effect of fatty acids, as the same concentration of PAM did not cause these effects, neither with nor without insulin (data not shown).

Previous studies have shown that excess glucose causes glucose toxicity that is manifested by the inhibition of glucose uptake and induction of insulin resistance in the muscles [42]. The toxicity of excess glucose is due to the activation of the hexamine pathway and production of glucosamine, which inhibits insulin-stimulated glucose uptake and subsequently glycogen synthesis [43]. Our observations that increasing amounts of glucose inhibit glucose uptake (Figure 4b and c) and glycogen synthesis (Figure 4d) are in agreement with these findings. Excess glucose did not affect the viability of the myotubes (Figure 4a), ruling out the possibility that the fall in glucose uptake was due to decreased viability of the myotubes. These findings stress the role of excess glucose in generating insulin resistance; it is therefore also noteworthy that neither PA nor insulin had any effect on glucose uptake or glycogen synthesis in myotubes that had been exposed to excess glucose.

## Conclusion

In the present study, we have been able to generate a suitable primary porcine cell model for studying both insulin and non- insulin-stimulated glucose uptake at physiological levels of insulin, as 0.1 nM insulin induces a significant increase in glucose uptake. We have shown that PA alone can improve glucose uptake but not glycogen synthesis and that during glucose uptake, PA probably competes with insulin in insulin-signaling. PA can neither stimulate glucose uptake nor glycogen synthesis in insulin-resistant cells generated by exposure to excess glucose. A potential role for PA may, thus, be stimulating glucose uptake in muscle cells at inadequate insulin concentrations, although the consequences of increasing glucose uptake without a concomitant increase in glycogen synthesis need to be studied further.

## Methods

### Materials and reagents

Human insulin, PA, PAM, dimethyl sulfoxide (DMSO), cyto B, essentially fatty acid-free bovine serum albumin (defatted BSA), glycogen, haematoxylin, chloral hydrate, cytosine arabinoside and antibiotics were all purchased from Sigma. Dulbecco’s Modified Eagle’s Medium (DMEM), fetal bovine serum (FBS), horse serum (HS) and phosphate buffered saline (PBS) were obtained from Life Technology. The WST-1 reagent was obtained from Boehringer Mannheim. Matrigel reagent was from Becton Dickinson, and the BCA kit was from Pierce Rockford. [1-3H] - 2-DOG was purchased from GE Healthcare and [1-^14^C]-D-glucose from PerkinElmer.

### Buffers and media

The proliferation growth media (PGM) consisted of DMEM containing 22 mM D-glucose, 10% FBS, 10% HS, 1% penicillin-streptomycin, 1.2% amphotericin B and 0.2% gentamycin. The first differentiation media (DM1) was as PGM but without HS and D-glucose was reduced to 6 mM. The second differentiation media (DM2) was as DM1, except for a reduction of FBS to 5% and addition of cytosine arabinoside to stop proliferation. The third differentiation media (DM3) was as DM2 but lacked serum. Hepes-buffered saline (HBS) pH 7.4 consisted of 20 mM Hepes, 140 mM NaCl, 5 mM KCl, 2.5 mM MgSO_4_ and 1 mM CaCl_2._ The storage medium was made up of PGM and 10% DMSO. Haematoxylin solution (100 mL) consisted of 5 g hydrated potassium sulphate, 0.01 g sodium iodate, 0.1 g haematoxylin dye, 5 g chloral hydrate and 0.1 g acetic acid to improve shelf-life of the solution.

The experimental media normally consisted of the stock media (2.5 mM D-glucose and defatted BSA) but where indicated, included different doses of insulin and/or glucose and fatty acids. Fatty acids were solubilized either in DMSO or ethanol. Ethanol was evaporated under a stream of liquid nitrogen while the final amount of DMSO in the experimental media was less than 0.1%. The experimental media with fatty acid was sterilized using a sterile filter and left overnight at room temperature to ensure efficient binding of defatted BSA to the free fatty acids. In this study, the fatty acid: BSA ratio was limited to 5:1. The amount of glucose, DMSO and defatted BSA in control samples was equivalent to that of treated samples, unless stated otherwise.

Media for glycogen analysis consisted of 5 mM D-glucose, 0.2 μCi [1-^14^C]-D-glucose and 0.1% defatted BSA in HBS. Glycogen carrier stock solution consisted of 0.5 g glycogen in 50 ml 30% KOH solution.

### Isolation and culture of primary porcine satellite cells

Primary satellite cells were obtained from the left *semi-membranosus* muscle of six weeks-old female pigs, weighing between 12–13.5 kg following a method described by Theil *et al.*[44]. Isolated cells were placed in storage medium and stored in liquid nitrogen until use. Cells were thawed in a 37°C water bath and seeded on 4% matrigel-coated plates containing PGM. After 2 days, cells were washed with 1xPBS, pH 7.4 (with Ca^2+^, Mg^2+^) and allowed to proliferate for 3–4 days to 80% confluence (Figure 5a and c). Cells were given DM1 until 100% confluent, and thereafter DM2 was given to stop proliferation and initiate differentiation. At this stage, the cells were denoted to be at their first day of differentiation. Cells were given DM2 until day 2 of differentiation, where fully developed myotubes were observed microscopically (Figure 5b and d). Myotubes were given DM3 16 h prior to experimental treatment. When the experimental treatment was administered for more than 4 h, DM3 was omitted. Cell culturing was performed at 37°C with 5% CO_2_ and at 100% humidity.

### Haematoxylin staining

Cells were rinsed twice with 1xPBS (without Ca^2+^, Mg^2+^) and fixed with ice-cold methanol for 5 min. The methanol was replaced by haematoxylin solution for 10 min, after which the dye was aspirated and cells were rinsed several times to discard unwanted stain. Cells were viewed for appropriate staining on a microscope at 100 × magnification.

### Viability test

After cell growth, differentiation and incubation with experimental media for 24 h, myotubes were rinsed with 1xPBS (with Ca^2+^, Mg^2+^) and WST-1 reagent was used as described by Okura *et al.*[45]. The generation of a formazan dye by cleavage of a tetrazolium salt present in the reagent reflects the amount of active cells present in culture. The absorbance of the culture media, which was corrected for blank readings in the presence of medium only, was measured at A_450-630_ using the Envision Multilabel Plate Reader model 2104 PerkinElmer.

### Protein determination

The protein concentration of myotube samples was determined using the bicinchroninic acid (BCA) assay as described earlier by Smith *et al.*[46].

### 2-deoxyglucose uptake

Cells seeded in 48-well plates were cultured as described earlier until the 2^nd^ or 3^rd^ day of differentiation. They were rinsed once with 1xPBS, pH 7.4 (with Ca^2+^, Mg^2+^) and incubated at given time points with experimental media (see figure legend). Cells were washed 3 × with HBS solution, and glucose uptake was carried out using [1-^3^H] 2-DOG as described by Klip *et al.*[47]. Unless stated otherwise, cells were incubated with 2-DOG for 30 min. Carrier-dependent glucose uptake was checked using [1-^3^H]2-DOG solution containing varying concentration of cyto B [40]. Samples were assayed for [1-^3^H] 2-DOG-uptake as disintegrations per min and normalized to the basal level of glucose uptake in control samples.

### Glycogen synthesis

Glycogen analysis was carried out on cells grown in 48-well plates as described by Al-Khalili *et al.*[36] with some modifications. On day 2 or 3 of differentiation, DM3 media was replaced with experimental media. Cells were rinsed afterwards with HBS solution and then incubated with fresh HBS solution containing 0.1 mM defatted BSA (and insulin where indicated) for 2 h. Media for glycogen analysis (containing [1-^14^C]-D-glucose) was added to the cells during the last 1.5 h of incubation. Cells were washed 3 times with ice-cold 1xPBS, pH 7.4 (without Ca^2+^, Mg^2+^) and lysed with 200 μL 30% KOH per well for at least 30 min. The cell lysate was transferred into eppendorf tubes, heated for 10 min at 96°C and 3 mg/ml of carrier glycogen was added to the cell lysate. A portion of the cell lysate was used for protein determination. Glycogen was precipitated by overnight incubation with 5 volumes of ethanol and 5% sodium sulfate at −20°C. The precipitated glycogen was collected by centrifugation at 20.800 × g for 5 min, and re-dissolved in distilled water. Radioactivity was measured in a scintillation mix as disintegrations per min and the values were corrected to the protein content of the cells.

### Statistical analysis

Statistical analyses were performed using the MIXED procedure in SAS version 9.2 (SAS Institute Inc., Cary, NC, USA). The results are presented as least square mean estimates (LSmeans) and the standard error of mean (SEM). When the main effects were significant, the least square mean estimates were separated by least significant difference (p < 0.05). All data are expressed as fold changes normalized to control samples.

### Ethics approval

Pigs used for the isolation of satellite cells were treated according to the Danish Ministry of Justice Law, no. 382 (June 10, 1987).

## Abbreviations

2-DOG: 2-deoxyglucose; BCA: Bicinchroninic acid; CLA: Conjugated linoleic acid; cyto B: Cytochalasin B; defatted BSA: Fatty acid-free bovine serum albumin; DM1: First differentiation media; DM2: Second differentiation media; DM3: Third differentiation media; DMEM: Dulbecco’s Modified Eagle’s Medium; DMSO: Dimethyl sulfoxide; FBS: Fetal bovine serum; GLUT: Glucose transporter; HBS: Hepes-buffered saline; HS: Horse serum; LSmeans: Least square mean estimates; PA: Phytanic acid; PAM: Palmitic acid; PBS: Phosphate buffered saline; PGM: Proliferation growth media; PPAR: Peroxisome proliferator-activated receptor; RXR: Retinoid-X-receptor; SEM: Standard error of mean.

## Competing interests

The authors declare that they have no competing interests.

## Authors’ contributions

BNC designed and performed experiments, analyzed data and wrote the manuscript. NO contributed to statistical analysis of data, discussion, review and editing of manuscript. LIH contributed to the conception of the project, discussion, interpretation, review and editing of the manuscript. JHN contributed to the conception of the project. JFY contributed to the experimental design, discussion, review and editing of manuscript. All authors read and approved the final manuscript.
